# Endometriosis in an indigenous African women population

**DOI:** 10.4314/ahs.v22i1.17

**Published:** 2022-03

**Authors:** Samuel Ohayi, Nnaemeka Onyishi, Sunday Mbah

**Affiliations:** 1 Department of Histopathology, Enugu State University Teaching Hospital, Parklane, GRA, Enugu; 2 Enugu State University of Science and Technology College of Medicine, Obstetrics and Gynaecology

**Keywords:** Endometriosis, indigenous African women, endometrial tissue, chronic pelvic pain, infertility, abnormal uterine bleeding

## Abstract

**Introduction:**

Endometriosis is the existence of endometrial tissue outside the endometrial cavity. It has high prevalence in women living in developed countries but is believed to be rare among indigenous African women.

**Objectives:**

This study aimed to determine the prevalence and characteristics of endometriosis in an indigenous African women population.

**Methods:**

Gynaecological specimens received and diagnosed as endometriosis in a teaching hospital's Histopathology laboratory over a 5-year period was retrospectively reviewed. Data obtained were analysed by simple statistical methods.

**Results:**

There were 25 diagnosed cases of endometriosis representing 0.9% of gynaecological specimens received in the period. Patients' average age is 38.4±8.4 years; peak age was 31- 40 years (n=10; 40%). Myometrium is the most common site (n=16; 64%), other sites include umbilicus and round ligament etc. Pelvic pain, 36% and irregular uterine bleeding, 28% are most common symptoms. There was primary and secondary infertility in 20% and 16% of cases respectively. The umbilical and suprapubic masses had symptoms that synchronised with the patient's menstrual cycle.

**Conclusion:**

Endometriosis has low prevalence in our population. Women presenting with chronic pelvic pain, infertility and menstrual disorders should be evaluated for endometriosis. Population-based study is required to further characterize the condition in our population.

## Introduction

Endometriosis, the presence of ectopic endometrial tissue, is a significant cause of gynaecologic consultation with attendant significant economic burden to society. 1,2 It is called adenomyosis (AM) when the ectopic tissue is found within the myometrium where it leads to smooth muscle hypertrophy, fibrosis and ultimately to uterine enlargement. When seen outside the uterus, the condition is called endometriosis (EM). A scheme based on macroscopic features classifies endometriosis into superficial peritoneal endometriosis (SUP), cystic ovarian endometriosis or endometrioma (OMA), and deeply infiltrating endometriosis (DIE).^3^,^4^ EM is complex and heterogeneous in several aspects including location, size, colour, depth of invasion and clinical manifestation. In fact, a 2000 work proposed designating only OMA and DIE as the ‘definite disease’ which the authors defined as ‘laparoscopically visible ovarian endometriomas of any size, endometriotic pelvic implants deeper than 5 mm, or any visible ectopic endometrial implants in the presence of ovarian or pelvic adhesions without another explanation.’^5^ It can affect pelvic and extra-pelvic structures extending as far as the anterior abdominal walls, intrathoracic structures and the brain. The condition is linked with uninterrupted episodes of ovulatory cycle and hormonal cycles. Endometriosis is the commonest cause of chronic pelvic pain in women of child-bearing age. Other symptoms include painful menstruation, dyspareunia and primary and or secondary infertility.^6^,^7^,^8^ These symptoms and their chronic nature are known to disrupt the lives of sufferers and also cause anxiety and depression in some individuals.

The most accepted pathogenetic mechanism is retrograde menstruation (Sampson's theory) in which products of menstruation are believed to flow in a retrograde manner through the fallopian tube into the pelvic cavity and then implant on pelvic structures.^9^ The second mechanism is the metaplastic theory which suggests that pelvic peritoneal surface epithelium undergoes metaplasia into endometrial tissue. Endometriosis of far removed sites including the intrathoracic structures is thought to be by vascular or lymphatic spread. However, the most plausible theory for intrathoracic endometriosis is that it originates from refluxed endometrial cells which then flow clockwise in the peritoneal fluid to implant on then penetrate the right leaf of the diaphragm to reach the right pleura. This may explain why about 90% of the thoracic endometriosis occur on the right side.^10^,^11^ Other putative theories of endometriosis include origin from embryonic stem cells and the induction theory.^12^,^13^ To make a histological diagnosis of EM, a pathologist must find any two of the following features on histological examination of surgical sample namely endometrial stromal tissue, endometrial glandular tissue and evidence of chronic haemorrhage (e.g. red blood cells and haemosiderin-laden macrophages) within or near a suspected endometrial tissue.^4^ In current practice, standard diagnosis of pelvic endometriosis is by pelvic laparoscopic visualization and surgery followed with histologic evaluation of biopsied tissue for confirmation of endometriosis.^5^ Basing diagnosis on gross laparoscopic visualization alone has the likelihood of missing microscopic deposits on the peritoneum as well as adenomyosis (DIE) each of which equally causes debilitating symptoms.^14^ For this reason, Bulum et al proposed that clinical diagnosis of endometriosis should only be by exclusion and should/span> be based on detailed history and symptoms.^4^

Endometriosis has a high prevalence in women living in developed countries and is reportedly the third most common cause of gynaecologic hospitalization in the USA.^15^,^16^ It has a prevalence of 2% to 10% in women of child-bearing age^17^ and up to 50% in infertile women. 18 However, it is believed to be rare among indigenous African women though studies on its prevalence in this population are few. Low prevalence in indigenous black women may be relative, possibly resulting from lack of facilities for laparoscopy for both diagnosis and surgery in our environment or poor reporting or both. Prevalence among African American women is reported to be similar to that of Caucasians.^16^ This finding suggests that there may be an actual low prevalence in African indigenous women. The differential prevalence rate between indigenous African women and African American women however, is believed to be due to non-genetic factors namely early onset of childbearing, frequent child bearing and recurrent pelvic inflammatory diseases which are typical of African indigenous women.^16^,^19^ This study aims to determine the prevalence and clinical characteristics of the condition in an indigenous African women population.

## Materials and Methods

This is a retrospective study of gynaecological specimens received in the Histopathology laboratory of a State University Teaching Hospital from January 1st 2014 to December 31^st^ 2018. A surgical specimen received in the laboratory is issued a laboratory identification number and registered in the laboratory's specimen register while making sure that it was put in the adequate amount of the appropriate fixative namely 10% formaldehyde. Thereafter each specimen is examined by a pathologist who takes blocks of the tissue in the process. These blocks are processed into slides, stained with haematoxylin and eosin and reported on by a pathologist. Afterwards, slides and paraffin tissue blocks are archived in designated places in the laboratory. Also, a hard copy of the pathology report is archive in the departmental library for results while an electronic copy is store in the departmental computer.

The study center is located in the capital city of a state which has a population of about 3.8 million according to 2016 estimates. The hospital receives patients from all parts of the state and also from neighbouring states. The hospital's histopathology unit receives most of its specimens from the relevant clinical units of the hospitals but also from other hospitals in the state.

For this research, laboratory request forms, laboratory specimen registers and histopathology reports all of which are archived in the laboratory were retrieved and relevant information about patients that had been diagnosed with endometriosis were extracted into a form designed for the purpose. This means that patients' clinical information not included in the laboratory request forms by the attending physicians were not captured for this study since patients' clinical notes were not part of the source of information. Data extracted included patient's age, presenting symptom/s, reproductive history, type of tissue submitted for histology and histological diagnosis among other relevant information. Information extracted was authenticated by matching relevant laboratory documents. Slides and tissue blocks were also retrieved. The slides were reviewed and where doubt existed about the histological diagnosis, fresh slides were made from tissue blocks and examined by a pathologist. Inclusion criteria include having a histologic diagnosis of endometriosis or adenomyosis in a submitted surgical specimen and having complete clinical data in the laboratory records.[V1] Data obtained were analysed using simple statistical methods.

Ethical approval was obtained from the Ethics committee of the hospital. Cases with incomplete patient information or in which doubt existed about the archived laboratory report but the specimen was lost were excluded from the study.

## Results

In the period under review, a total of 2778 gynaecological specimens were received in our histopathology laboratory. Out of these, 25 (0.9%) were diagnosed as ectopic endometrial tissue and having met the inclusion criteria were included in the study. These consist of 9 (0.3%) cases of endometriosis and 16 (0.6%) cases of adenomyosis corresponding to a proportion of 0.003 (95% CI, 0.002 – 0.006) and 0.006 (95% CI, 0.003 – 0.009) respectively. Table 1 shows the distribution of the subjects' ages at menarche and at the onset of symptoms.

**Table 1:** Age characteristics of patients

Age attained menarche						
Age (in years)	11	12	13	14	15	16
No. of patients	2	8	7	3	3	2
Age at onset of symptoms						
Age (in years)	21 – 30	31 – 40	41 – 50	51 – 60		
No. of patients	6	10	7	2		

**Figure 1:**
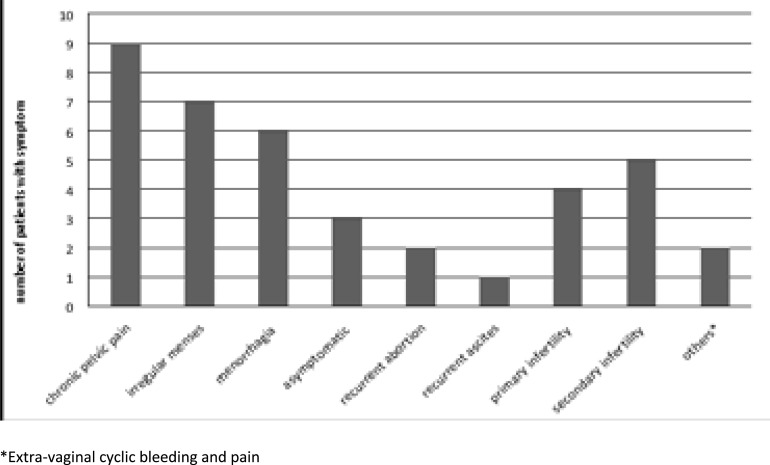
Distribution of patients' symptoms

The average age and median age of patients at diagnosis was 38.4±8.4 (95% CI, 34.9 – 40.9) and 39.0 years respectively while the peak age of occurrence was 31 - 40 years (n=10; 40%). Average age and peak age at menarche was 13.1±1.3 years (95% CI, 12.6 – 13.6) and 12 – 13 years respectively. The myometrium is the most common site (n=16, 64%; 95% CI, 0.003 – 0.009) followed by the ovary (n=5; 20%). The umbilicus, round ligament, broad ligament and a suprapubic scar constituted 1 (4%) respectively. Figure 1 contains an illustration of symptoms of the patients at presentation.

The most common symptoms were chronic pelvic pain, (n=9; 36%) and irregular uterine bleeding, (n= 7; 28%) which correspond to a proportion of 0.36 (95% CI, 0.17 – 0.54) and 0.28 (95% CI, 0.10 – 0.46) respectively while recurrent ascites was the least (n=1; 4%). There was associated primary and secondary infertility in 20% and 16% of cases respectively. Recurrent abortion occurred in 2 (8%) of cases. The umbilical and suprapubic lesions had symptoms that synchronised with the patient's menstrual cycle namely bleeding and tender enlargement respectively. Frequency of symptoms exceeded number of subjects because some subjects had multiple symptoms.

## Discussion

Endometriosis is a chronic gynaecologic disease which affects women of reproductive age. Though a benign condition, it is associated with distressing consequences ranging from debilitating chronic symptoms of the primary disease to its attendant complications. In this study, the average age and peak age of patients at diagnosis of 38.4±8.4 years and 31 – 40 years respectively is comparable to that reported in other works.^20^,^21^ Similarly, the median age in our patients compares with that reported from another study.^22^ The average age and peak age at which women in this study attained menarche is comparable to that reported from Rumania.^23^ The prevalence of endometriosis among our population is low according this study. The rate of 0.9% in women who underwent gynaecologic surgeries is comparable with other Nigerian studies that recorded 0.4% in women undergoing gynaecologic surgeries^24^ and 1.8% in women being treated for infertility.^25^ It is also comparable with findings in black African women of South Africa.^19^ It is however in contrast with higher prevalence rate reported among Caucasian women.^4^,^15^,^16^,^20^ In addition to reporting a low prevalence among Ugandan women, Somigliana et al 25 also reported an inverse relationship between high fertility rate, frequent teen-age pregnancy and protracted breast-feeding and the prevalence of the disease. Some workers believe that the reported low prevalence in indigenous African women may not be actual but due to underreporting, lack of state of the art diagnostic equipment including MRI diagnosis, and lack of trained experts for surgical diagnosis and therefore lack of access to specialised care, and as such are of the view that the prevalence rate may increase when these conditions change.^27^,^28^ The drawbacks associated with diagnosis of endometriosis and adenomyosis based on direct visualization with histological confirmation on removed tissues include the uncertainty about the true prevalence of the disease, delay in diagnosis and treatment among others. This has stimulated research interest into finding biomarkers in various body tissues and substances including blood, urine, endometrialand menstrual fluid for the condition. The search for biomarkers continues as none with sufficient sensitivity and specificity has been found.^29^,^30^

The most common symptom seen in the patients in this study is non-cyclical chronic pelvic pain (36%), abnormal menstrual bleeding (32%) and dysmenorrhoea (32%). This is similar to findings from other studies.^20^,^21^,^22^ Two of the studies^21^,^22^ however reported very high rates of 71.1% - 81.8% and 68% respectively for dysmenorrhoea. The rate of deep dyspareunia in this study is also comparable to those reported in other studies.^21^,^22^ Gastointestinal symptoms were rare in this study in contrast with a rate of 30.1% and 43.6% reported elsewhere for OMA and DIE respectively.^21^ This may be because the attending physicians did not include the information in the laboratory request forms which was the only source of patients' information for this study. Patients in this study who had endomtriotic masses in uncommon sites also presented with some rare and unique symptoms not commonly reported in literature. However, such symptoms synchronising with patients' menstrual cycle have been reported in some studies.^26^,^27^,^31^,^32^ These symptoms which were synchronous with menstrual bleeding include bleeding from endometriotic site and tenderness with increase in size of an endometriotic mass/site. Diagnosis of each of the casepresenting with these symptoms was aided by the presenting symptom. Primary and secondary infertility was present in 20% and 16% of the women in this study. This is comparable to reports by other workers^21^,^22^ but lower than 47.5% reported from another study of 1000 women who also had other different benign gynaecological conditions.^20^ The most common site of involvement of endometriosis in our study is the myometrium a finding that contrasts other studies which reported the ovary as the most commonly affected site.^20^,^21^,^22^ Among our study population, endometriosis also occurred in some rare sites, a finding that is not common with most studies.

## Conclusion

Endometriosis appears to have low prevalence in our population. Also, in addition to the regular sites, it occurs in rare sites. Women presenting with chronic pelvic pain and infertility and menstrual disorders should also be evaluated for endometriosis. So also should women presenting with relevant symptoms in unusual sites including surgical scars and umbilicus that synchronise with menstruation. Population-based study is required to properly determine the actual prevalence of the condition and its characteristics in indigenous African women. Given the debilitating nature of its symptoms and its association with fertility problems, there is need to invest in training specialized personnel and in providing state of the art equipment for timely diagnosis of the condition in our society.
